# Olaparib and Doxorubicin Co-Loaded Polypeptide Nanogel for Enhanced Breast Cancer Therapy

**DOI:** 10.3389/fbioe.2022.904344

**Published:** 2022-05-02

**Authors:** Yanhong Liu, Meiyan Wang, Wanru Liu, Jili Jing, Hongshuang Ma

**Affiliations:** ^1^ Center for Reproductive Medicine, Center for Prenatal Diagnosis, First Hospital, Jilin University, Changchun, China; ^2^ Department of Rheumatology and Immunology, First Hospital, Jilin University, Changchun, China

**Keywords:** olaparib, polypeptide, reduction-responsive, co-delivery, molecular targeted therapy

## Abstract

Although great progress has been made in improving the efficacy of cancer treatment through combination treatment using drug agents, there are still challenges in improving the efficiency of drug delivery. In this study, olaparib and doxorubicin were co-loaded on disulfide bond cross-linked polypeptide nanogels for the treatment of breast cancer in mouse models. Under stimulation of a high glutathione environment in cancer cells, the drug is quickly released from the nanogel to target cancer cells. In addition, compared with free drugs and single-drug-loaded nanogels, dual-drug- co-loaded nanogels exhibit the best anti-cancer effect and demonstrated excellent biological safety. Therefore, the co-delivery of olaparib and doxorubicin through polypeptide nanogels presents good prospects for application as anti-cancer treatment.

## Introduction

Breast cancer has surpassed lung cancer as the most common malignant tumor in women in the world. Its incidence accounts for about 24.5% of female tumors and is also the fifth leading cause of cancer deaths in the world, with 685 thousand people dying annually (Sung et al., 2021). In recent years, the mortality rate of breast cancer has increased. Chemotherapy, together with surgery or radiation therapy, remains the primary treatment for breast cancer. Nontargeted delivery of chemotherapeutic medications, on the other hand, has a significant impact on normal cells and, to a large extent, limits the drug dose reaching tumor cells, causing the reduction of the drug’s therapeutic effect (Liu et al., 2021). At present, clinically used drugs for advanced breast cancer include anthracyclines, taxanes, capecitabine, doxorubicin (DOX), and epirubicin (Aebi et al., 2014). However, chemotherapeutic drugs are highly toxic, the maintenance therapy administration cycle is longer, patient compliance is poor, there are many adverse reactions, and drug resistance easily develops (Spugnini et al., 2011; Dasari and Tchounwou, 2014). Therefore, an ideal therapeutic drug should have the characteristics of efficacy, low toxicity, and convenient use. Poly (ADP-ribose) polymerase (PARP) inhibitors are targeted small-molecule drugs that meet current clinical requirements; they have low toxicity, and do not easily develop drug resistance (Anders et al., 2010; He et al., 2010; Yap et al., 2011).

The target of PARP inhibitors is a protein modification enzyme that can catalyze the transfer of ADP-ribose in NAD + molecules to the amino acid residues of proteins and catalyze the synthesis of polyadenylic acid. Phosphoribose (PAR) modifies protein and regulates protein function (Lord and Ashworth, 2017). There are 17 members in the PARP family. Currently research has focused on PARP1/2. These two enzymes can catalyze the poly ADP ribosylation modification of proteins, participate in DNA damage repair, and play a role in the occurrence and development of a variety of tumor diseases. PARP1/2 is required for the repair of DNA single-strand breaks through base shear repair, and the repair of these break sites is the cause of resistance to radiotherapy and chemotherapy, such as that to alkylating agents (Dantzer et al., 1999). A large number of studies have shown that in tumor cells, PARP is generally highly expressed, making tumors prone to tolerance to treatment. Therefore, inhibition of PARP can reduce tumor tolerance, and PARP has also become a new target for tumor therapy.

As the first PARP inhibitor on the market, olaparib (Ola) has been approved by the United States Food and Drug Administration for the treatment of advanced ovarian cancer. At the same time, studies have shown that Ola also has a good therapeutic effect on a variety of breast cancers (Robert et al., 2017; Griguolo et al., 2018).

Smart drug delivery systems can overcome biological obstacles and improve biodistribution in the body and have shown great potential in cancer chemotherapy (Zheng et al., 2020; Chen et al., 2021; Wang et al., 2021; Zheng et al., 2021). These systems can greatly reduce the toxicity of chemotherapy drugs, prolong blood circulation time, enhance the sustained release of drugs, target specific tumors, and control drug release at targeted sites, thereby improving the effect of anti-tumor treatment and reducing side effects. Recently, disulfide bond-cross-linked block copolymers based on amphiphilic peptides have attracted much attention due to their biocompatibility, biodegradability, and precise secondary conformation (Liu et al., 2011; Li et al., 2014; Kong et al., 2016; Feng et al., 2021; Ma et al., 2021). This copolymer is very suitable for the delivery of anticancer drugs. Generally, amphiphilic polypeptides are composed of polyethylene glycol (PEG) and hydrophobically derived polypeptides. The hydrophobic core can be used as a reservoir and can also encapsulate hydrophobic drugs (such as paclitaxel, DOX, and amphotericin B), or it can be covalently combined with drugs (such as paclitaxel and DOX) to form the polymer.

In previous studies by our group, a series of nanogels based on methoxy poly (ethylene glycol)-poly (L-phenylalanine-co-L-cystine) (mPEG-(LP-co-LC)) were developed for tumor drug delivery. In this study, we prepared mPEG-(LP-co-LC) nanogels and used them to design a local Ola and DOX co-delivery system to treat breast cancer using a mouse model. The results show that the drug-loaded nanogel has a good therapeutic effect on tumors and exhibited better biological safety than free drugs.

## Materials and Methods

### Materials

Methoxy poly (ethylene glycol) (mPEG, M_n_ = 5,000) was purchased from Sigma-Aldrich without further purification. Aminomonomethoxypoly (ethy1ene glycol) (mPEG-NH_2_) was synthesized as previously described (Monfardini et al., 1995). Tetrahydrofuran (THF) was refluxed and dried with sodium prior to use and distilled under reduced pressure. L-cystine (LC) and L-phenylalanine (LP) were purchased from Gil Biochemical Co., Ltd (Shanghai, China), LP-NCA and LC-NCA were synthesized according to the previous method of our group (Ding et al., 2011; Yu et al., 2014), and Ola was purchased from Afghanistan Latin. The 4T1 cells were purchased from BNCC (Beijing). Animals were purchased from Vital River (Beijing), all animal experiments in this work were approved by the Institutional Animal Care and Use Committee of Jilin University (20200105).

### Synthesis of Disulfide Bond Cross-Linked Polyethylene Glycol-Polyamino Acid Copolymer

The disulfide bond crosslinked polyethylene glycol-polyamino acid copolymer was synthesized by the one-step ring opening polymerization (ROP) of LP-NCA and LC-NCA using PEG-NH_2_ as a macroinitiator (Li et al., 2018; Feng et al., 2021). Normally, 15.0 ml of dry DMF is added to a dry bottle containing mPEG-NH_2_, LC-NCA, and LP-NCA. The polymerization reaction was conducted in the bottle for 3 days and was then settled in excess ether. The obtained product was washed twice with ether and dried under vacuum at room temperature for 24 h.

### Preparation of Drug-Loaded Nanogels

First, 50 mg mPEG-(LP-co-LC) was dissolved in 1 ml DMF, and then was slowly introduced dropwise into 2 ml 10% PBS to prepare nanogels. The following quantities were added: 3 mg Ola, 5 mg DOX, 1 mg DOX and 3 mg Ola respectively to prepare mPEG-(LP-co-LC)/Ola nanogel (Ola NG), mPEG-(LP-co-LC)/DOX nanogel (DOX NG), and mPEG-(LP-co-LC)/DOX/Ola nanogel (DOX/Ola NG). The mixture liquid was stirred overnight, dialyzed in deionized water for 8 h (molecular weight cut-off (MWCO) = 7,000 Da). The solution was filtered and lyophilized.

The standard curve approach was used to measure the drug loading content (DLC) and drug loading efficiency (DLE). The DLC and DLE of drug-loaded nanogels were calculated according to Eqs. 1, 2, respectively.

$$\text{DLC}\left(\text{\%}\right)=\frac{Weight\;of\;Drug\;in\;Nanogel}{Weight\;of\;Drug-Loaded\;Nanogel}\times 100\%$$ (1)

$$\text{DLE}\left(\text{\%}\right)=\frac{Weight\;of\;Drug\;in\;Nanogel}{Weight\;of\;Feeding\;Drug}\times 100\%$$ (2)

### Drug Release

The dialysis method was used to detect the release of different nanogels in phosphate buffered saline (PBS) containing glutathione (10 mM) and without glutathione. The specific operation was as follows: 10.0 mg DOX NG, 10.0 mg Ola NG, and 10.0 mg DOX/Ola NG, respectively, were dissolved in 1 ml of the corresponding release medium, which was then introduced into the dialysis bag (molecular weight cut-off = 1,000 Da). The end of the dialysis bag was sealed and placed in 30 ml of the corresponding release medium at 37°C, and the release experiment was started at a continuous vibration rate of 80 rpm. At a predetermined time, 1 ml of dialysate was removed and replaced with an equivalent amount of fresh release fluid. HPLC was used to determine the absorbance of 10 μL of the solution at a wavelength of 276 nm UV and the accumulation of the release of Ola was calculated. A microplate reader was used to test the absorbance of 200 μl of the solution at the 488 nm UV wavelength, and the cumulative release of DOX was calculated.

### Cytotoxicity Determination

The relative cytotoxicity of free drugs and drug-loaded nanogels was evaluated in 4T1 cells using the CCK-8 detection method. Cells were seeded approximately 2000 cells per well in a 96-well plate, each well containing 200 μL of complete RPMI-1640. The plates were incubated for 12 h at 37 °C in a 5% CO_2_ environment, then the medium was removed and different amounts of free drugs and drug-loaded nanogel solutions were added. After incubation for another 48 h, the medium was discarded and added to a complete medium containing 10% CCK-8 and incubated for 2 h to perform the CCK-8 assay on the cells.

### *In vivo* Anti-tumor Efficacy of Nanoparticles in 4T1 Tumors

The 4T1 tumor model was prepared by subcutaneous injection of 4T1 cells (1×10^6^ cells/mouse) into the left abdomen of BALB/c mice (female, 6 weeks, 16–18 g). When the tumor volume reached 80–100 mm^3^, PBS, Ola/NG (Ola: 50 mg/kg), Free DOX/Ola, DOX NG (DOX: 4 mg/kg), and DOX/Ola NG (Ola: 50 mg/kg; DOX: 4 mg/kg) were injected intravenously into the 4T1 tumor-bearing mice. Body weight and tumor volume were measured every other day. The tumor volume (V) was calculated as follows:

$$\text{V}=\frac{a\times b^{2}}{2}$$ (3)

where (a) and (b) are the long and short axes of the tumor measured with a caliper. After treatment, the tumors were collected and photographed.

## Results and Discussion

### Synthesis and Characterization of Methoxy Polyethylene Glycol-Polypeptide Nanogel

**FIGURE 1:**
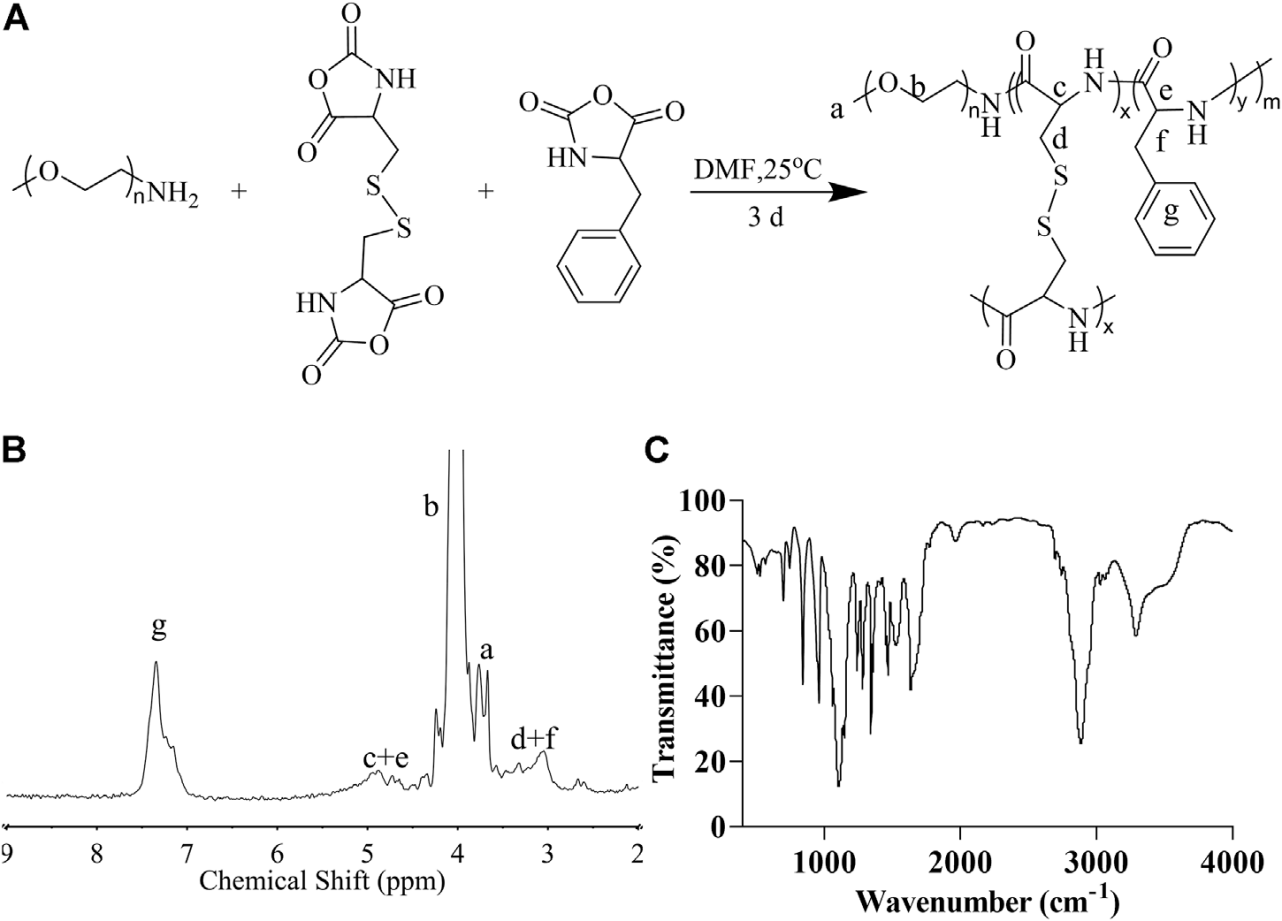
The synthesis and characterization of mPEG-P (LP-co-LC). The synthesis formula **(A)**, ^1^H NMR **(B)**, and FT-IR **(C)** of mPEG-P (LP-co-LC).

First, we synthesized cystine NCA and phenylalanine NCA and then used mPEG_5k_-NH_2_ as an initiator to conduct the ROP of amino acid NCA to obtain mPEG-(LP-co-LC) (Figure 1A). In this smart nanogel, cystine blocks are used to form a cross-linked structure, which could be degraded by glutathione (GSH), and phenylalanine blocks are used as a reservoir for the drug, which was encapsulated in the core of the nanogel through the interaction between the phenyl rings. As illustrated in Figure 1B, the ^1^H NMR exhibited a proton resonance signal in the benzene ring of the LP unit (C_6_H_5_-, 5H) at 7.26 ppm (g). The proton resonance of the polypeptide backbone (-C(O)CH(CH_2_C_6_H_5_)NH-, 1H and -C(O)CH(CH_2_S-)NH-, 1H) peaked at 4.85–4.31 ppm (c + e). The resonances of the methylene and terminal methoxy protons in mPEG (-CH_2_CH_2_-, 4H and CH_3_-, 3H) were attributed to the signals at 3.91 (b) and 3.58 ppm (a), respectively. At 3.26-2.94 ppm (d + f), the methylene protons in the side groups of the polypeptide showed distinctive signals (C_6_H_5_CH_2_-, 2H and -SCH_2_-, 2H). As shown in Figure 1C the FT-IR spectrum of mPEG-P (LP-co-LC) further demonstrated the successful synthesis of the material through the typical absorption at 1,636 (ʋ_C=O_) and 1,468 cm^−1^ (ʋ_C(O)-NH_).

### Preparation and Characterization of Nanogels Loaded With Doxorubicin and Olaparib

DOX NG, Ola NG, DOX/Ola NG were obtained using the nanoprecipitation method, the DLCs of DOX NG, Ola NG, and DOX/Ola NG were 8%, 4.75%, and 1%/3%, respectively. The DLE of DOX NG, Ola NG, and DOX/Ola NG were 48%, 25.65% and 51%/23%, respectively. The results showed that we had successfully prepared nanogels with high drug loading efficiency. A higher drug loading rate is helpful for better drug delivery and release and can reduce multidrug resistance caused by large doses (Blanco et al., 2015). The amphiphilic polyamino acid copolymer can directly embed the small-molecule hydrophobic drugs DOX and Ola into the hydrophobic core after self-assembly in the aqueous solution, effectively improving the solubility and stability of the drug, and the internal disulfide bond increased the stability of the cross-linked core.

**FIGURE 2:**
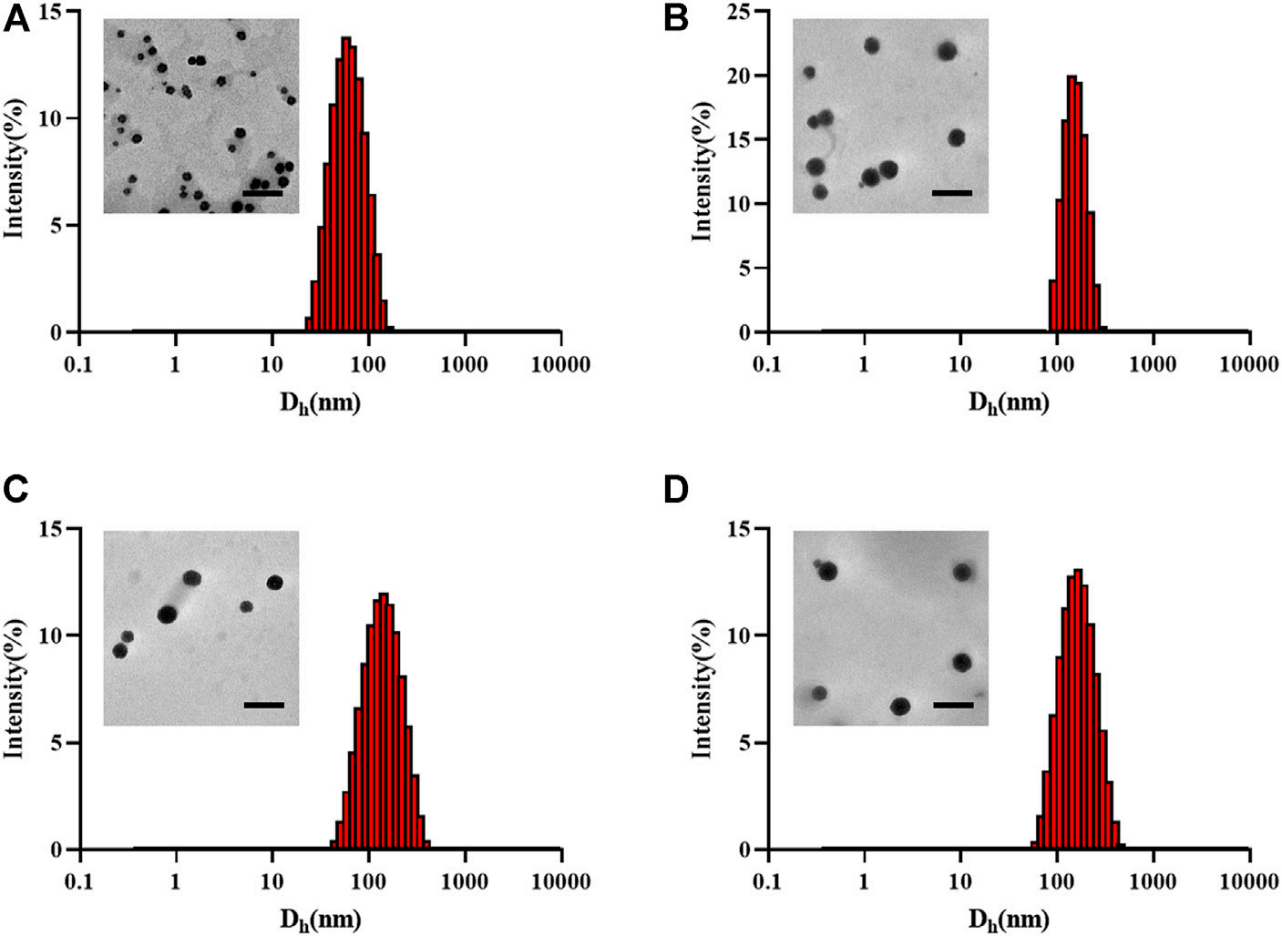
Physical characterization of nanogels. The TEM and hydrodynamic diameter detected by DLS of unloaded nanogel **(A)**, DOX NG **(B)**, Ola NG **(C)**, and DOX/Ola NG **(D)**. The scale bar is 500 nm.

The particle sizes of the nanogels were characterized by TEM and DLS. As shown in Figure 2, the diameter of the unloaded nanogel is approximately 65.86 nm, DOX NG, Ola NG and DOX/Ola NG are 150, 156.8, and 175.3 nm, respectively. As the types of loaded drugs increased, the particle size of the nanogel also increased. This was similar to the TEM image. The particle size of the TEM image was smaller than that measured by DLS due to the shrinkage of the nanogel (Yan et al., 2012).

### *In vitro* Drug Release

**FIGURE 3:**
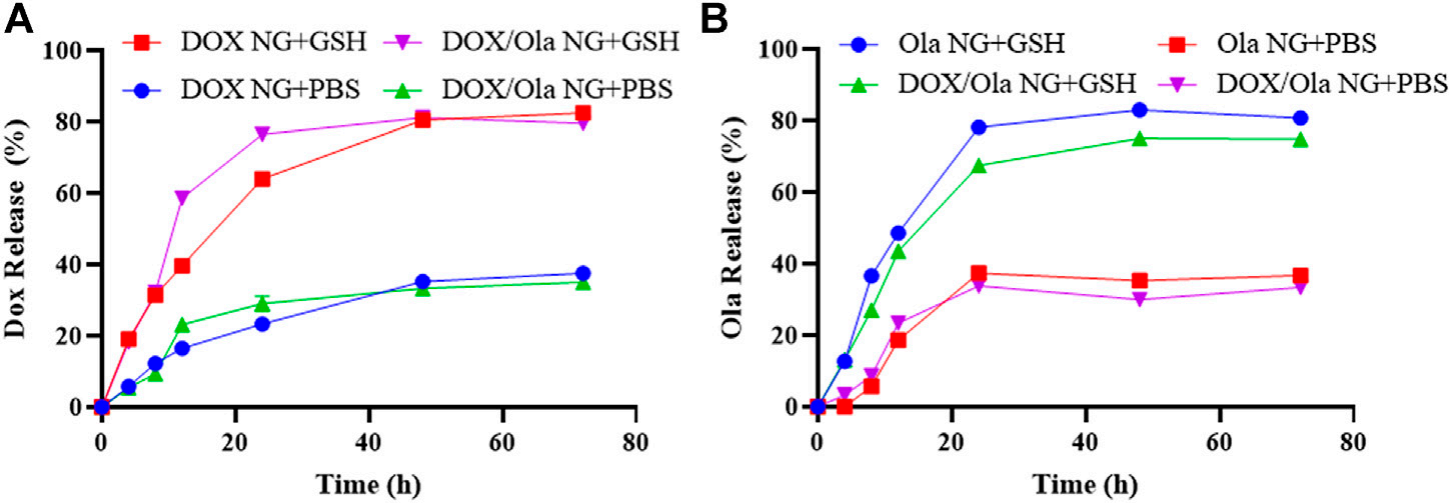
*In vitro* release of nanogels. The release of DOX **(A)** and Ola**(B)** in PBS of pH 7.4 or 7.4 with 10.0 mM GSH at 37°C.

The ratio of drug release is shown in Figure 3. In PBS medium without GSH, the cumulative amounts of DOX released from DOX NG and DOX/Ola NG within 24 h were 23.29% and 29.16%, and Ola released from Ola NG and DOX/Ola NG were 37.35% and 33.81%. At the same time, in the simulated intracellular microenvironment (PBS containing 10.0 nM GSH), the cumulative amount of DOX released DOX NG and DOX/Ola NG increased to 63.92% and 76.43%, respectively, and the Ola released from Ola NG and DOX/Ola NG increased to 78.09% and 67.46%. The results showed that when the release reached a plateau after 72 h, the nanogel could selectively release the molecularly targeted drug Ola and the chemotherapeutic drug DOX to the cells (Feng et al., 2021).

### Endocytosis and Toxicity

**FIGURE 4:**
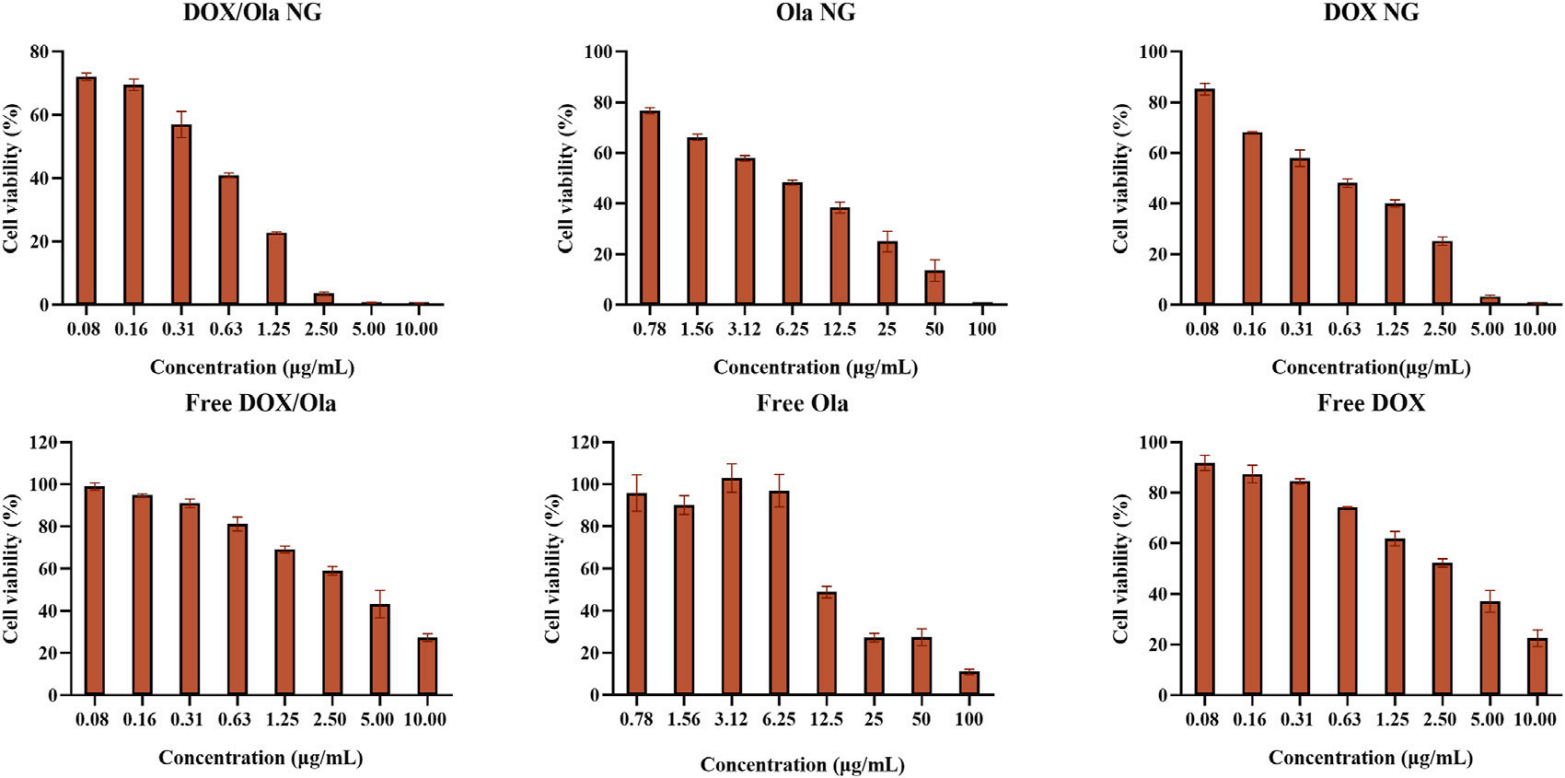
Cytotoxicity experiment. The *in vitro* cytotoxicity of nanogels after incubation with 4T1 cells for 48 h. The statistical data are presented as a mean ± SD (*n* = 3).

CCK-8 tested the 48-h cytotoxicity of the nanogels and free drugs on 4T1 cells. As shown in Figure 4, drug-loaded nanogels had a greater cytostatic effect than the free drug. The IC_50_ values of DOX NG, Ola NG, and DOX/Ola NG were 0.52 μg/ml, 5.12 μg/ml, and 0.34 μg/ml, respectively. DOX/Ola NG showed the best cytotoxic effects, and Free DOX/Ola achieved better cytotoxicity than Free DOX. This demonstrated that Ola could enhance the therapeutic effects of DOX, which has also been described for ovarian cancer (Bixel and Hays, 2015). Compared to free Ola, Ola NG had a superior inhibitory effect, proving that Ola could enhance the therapeutic effects by improving delivery efficiency, which has also been mentioned in previous studies (Mazzucchelli et al., 2017). DOX NG also has a better inhibitory effect than Free DOX. This is because free drugs have a faster metabolic rate in the cell, and nanogels have a better inhibitory effect due to endocytosis. In past studies, researchers have studied the synergistic effect of doxorubicin and olaparib, and proved that DOX and Ola have a good synergistic effect on cancer cells (Eetezadi et al., 2018; Miyamoto et al., 2019; Perez et al., 2020). Therefore, the drug effect equation based on Chou-Talalay (Zhu et al., 2010). We calculated the combination index (CI) at a Dox:Ola ratio of 1:3, and the CI value was 0.85, which indicating synergy.

**FIGURE 5:**
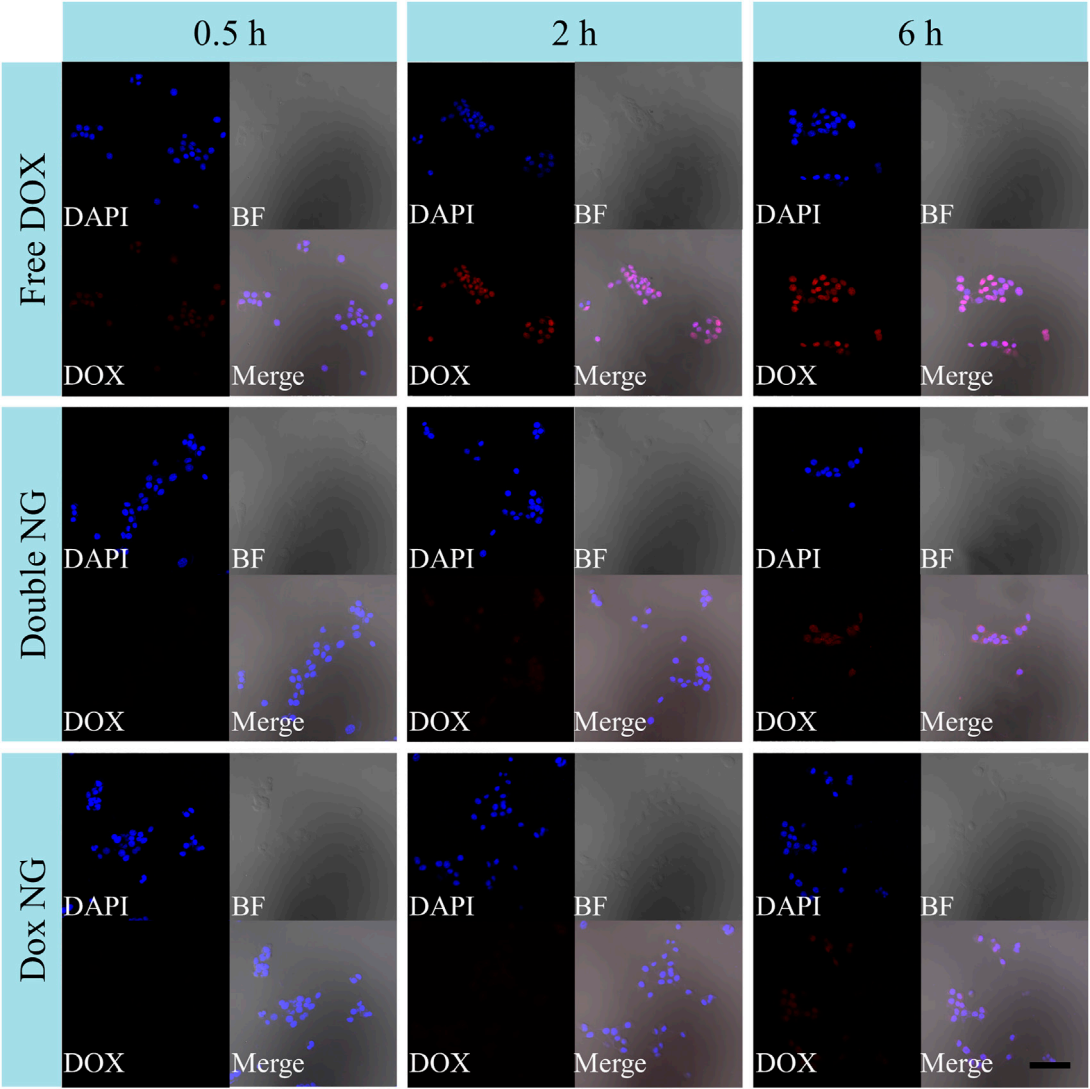
Endocytosis experiment. The CLSM microimaging analyses for cell endocytosis of Free DOX, DOX/Ola NG, and DOX NG. The blue channel is DAPI nuclear staining, the red channel is DOX staining, and the gray channel is brightfield. The scale bar is 100 μm.

As shown in Figure 5, the uptake of drug-loaded nanogels in 4T1 cells was tested by CLSM to confirm whether reduction-responsive nanogels are effective in transporting DOX into cells. The 4T1 cells were co-cultured with the drug-loaded nanogel and Free DOX for 0.5, 2, and 6 h (10.0 mg/L DOX). As expected, with increasing co-culture time, the fluorescence intensity in the cells increased accordingly. Several researchers including this research group have reported that due to the self-quenching effect of DOX, at the same concentration, Free DOX has stronger fluorescence than DOX loaded in nanoparticles (Sun et al., 2009; Wang et al., 2010). Therefore, the enhanced fluorescence intensity in 4T1 cells is due to high-efficiency endocytosis and degradation of drug-loaded nanogels, resulting in more DOX release in cells.

### Evaluation of the Anti-tumor and Safety of Drug-Loaded Nanogel on Orthotopic Breast Cancer Model

**FIGURE 6:**
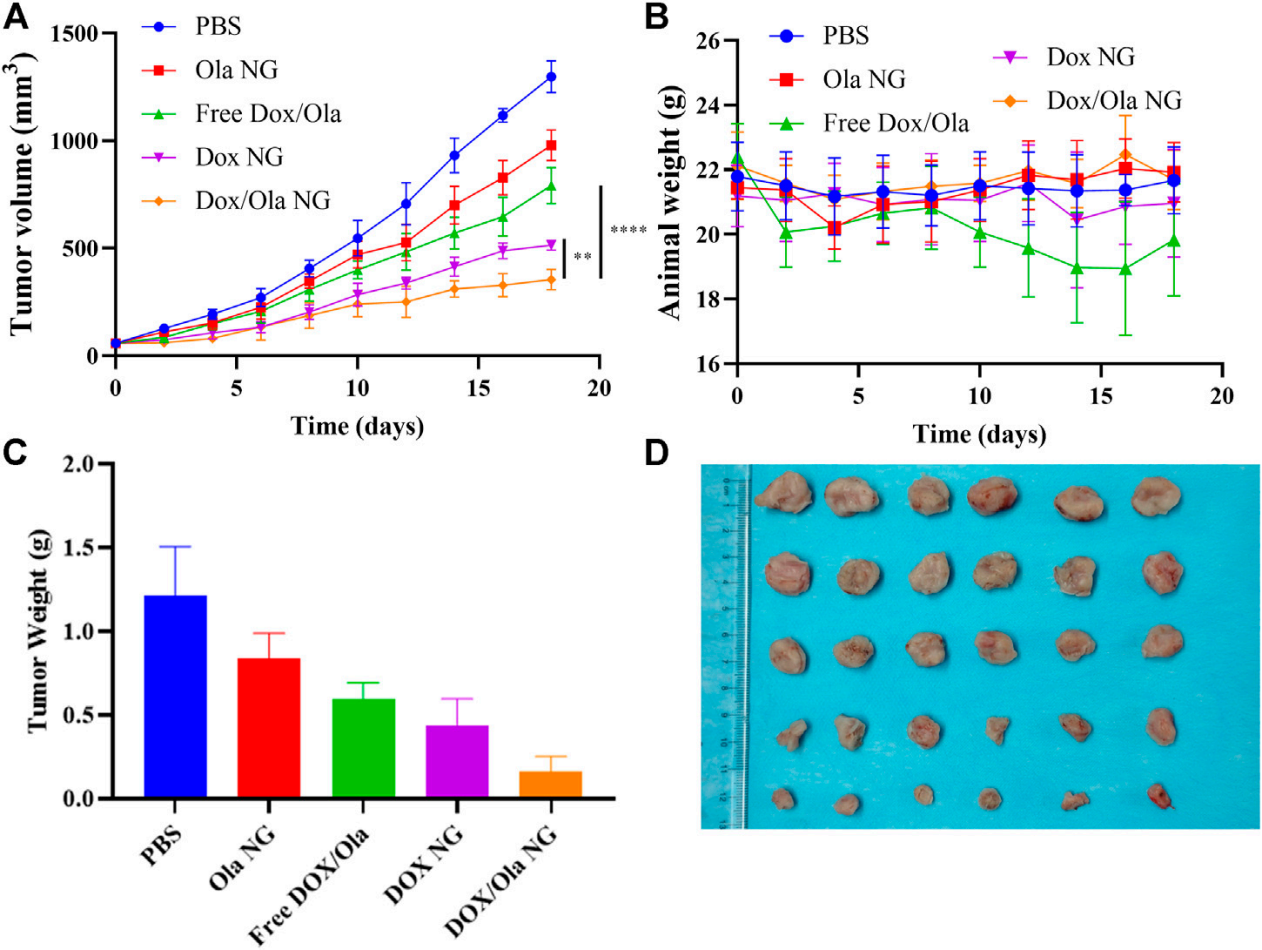
*In vivo* tumor suppression effect. The changes of tumor volume **(A)**, body weight **(B)**, tumor weight **(C)**, and tumor tissue photos **(D)** of 4T1-bearing BALB/C mice treated with PBS, Ola NG, Free DOX/Ola, DOX NG, and DOX/Ola NG. Each set of data is represented as mean ± SD (*n* = 6).

**FIGURE 7:**
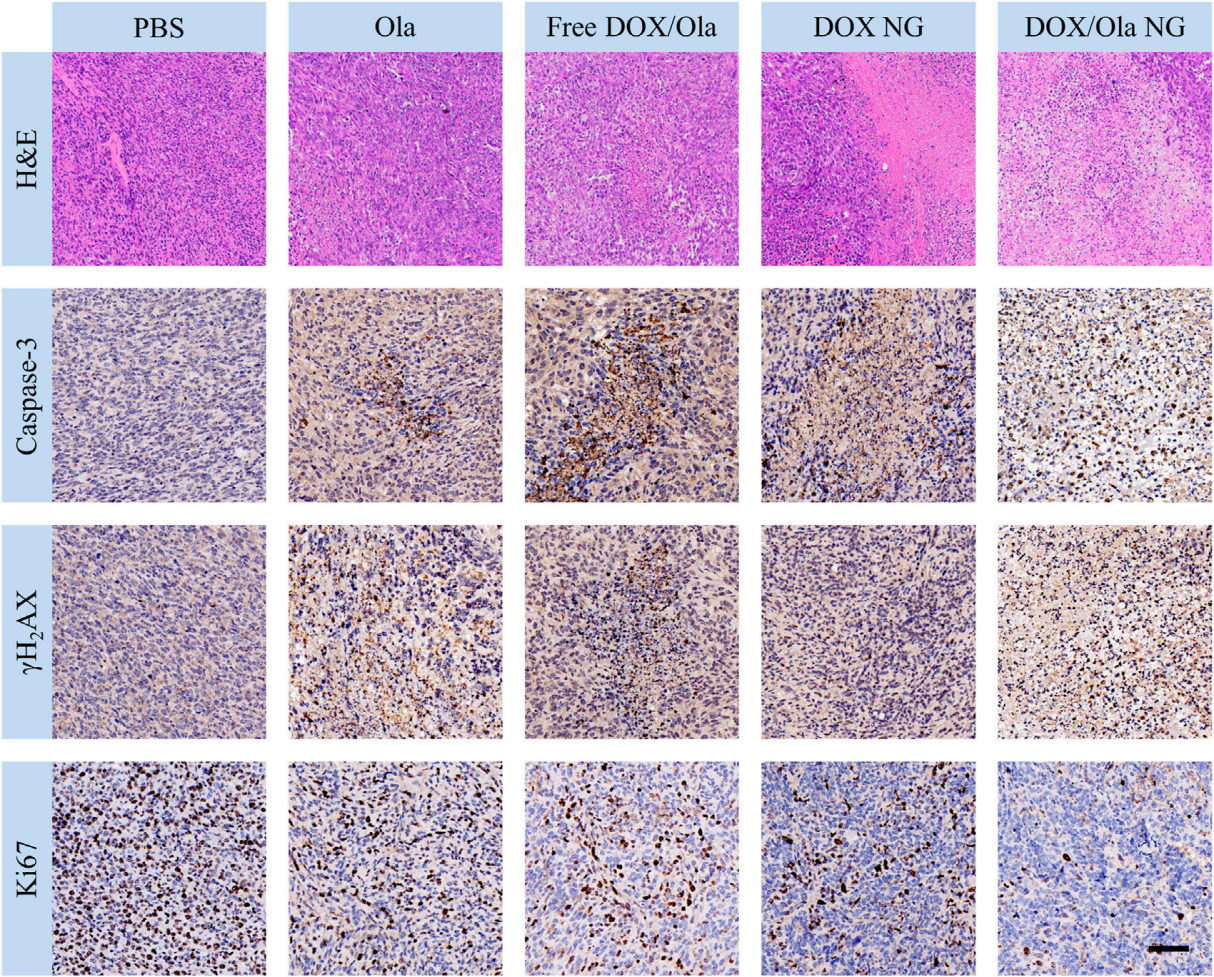
The immunohistochemical analysis of caspase-3, γH_2_AX, and Ki67 expression and H&E of tumor tissue. The scale bar is 100 μm.

To evaluate the therapeutic effects of the drug *in vivo*, a mouse orthotopic breast cancer model was constructed by injecting 1×10^6^ 4T1 cells into the abdomen nipple of mice. On the 10th day after injection, the tumor-bearing animals were randomly divided into four groups, and every 3 days they were treated with PBS, Ola NG, Free DOX/Ola, DOX NG, and DOX/Ola NG. The Ola dose was devised to be 50 mg/kg, and DOX dose was devised to 4 mg/kg. As shown in Figure 6A, by measuring and observing the tumors of mice during the treatment cycle, it was found that the tumor volume changed slightly from ∼60 to 354 mm^3^ after treatment with DOX/Ola NG. DOX NG haf an effect second only to DOX/Ola NG, and the tumor volume grew to 513 mm^3^ after treatment. The free drug and Ola NG also had a certain inhibitory effect on tumor growth. During the experiment, the tumor in the free drugs group grew to approximately 979 mm^3^. The free drugs showed a similar anti-tumor effect as Ola NG. As shown in Figure 6B, the free drug group showed a decrease in body weight, and the other groups did not show a significant change in body weight, which was due to the toxicity of DOX in the free drug. As shown in Figure 6C, D, after removing the mouse tumors, the DOX/Ola NG-treated mouse tumor had the smallest volume and the lowest weight. Furthermore, as shown in Figure 7, the tumor slices treated with DOX/Ola NG had the largest necrotic area compared to the PBS group, which proved the best tumor suppression efficiency of DOX/Ola NG.

At the same time, we performed immunohistochemical staining on tumor tissues to further confirm the inhibitory effect of drug-loaded nanogels and drugs on tumors. We performed an immunohistochemical characterization of tumor caspase-3, Ki67, and γH_2_AX proteins. It is well known that caspase-3, Ki67, and γH2AX are markers of apoptosis, cell proliferation, and DNA damage, respectively (Porter and Jänicke, 1999; Mah et al., 2010; Pathmanathan and Balleine, 2013). As shown in Figure 7, compared to the PBS group, the DOX/Ola NG group showed the largest apoptotic area and the best proliferation inhibitory effect, while Ola NG caused the smallest apoptotic area and the worst proliferation inhibitory effect, which was consistent with tumor suppression. It is consistent with the results of H&E staining. In the free drug group, the expression of γH_2_AX was found in the Ola NG and DOX/Ola NG groups and both Ola NG and DOX/Ola NG groups had a larger area of expression, which proved that drug delivery through nanogels has a better delivery effect.

**FIGURE 8:**
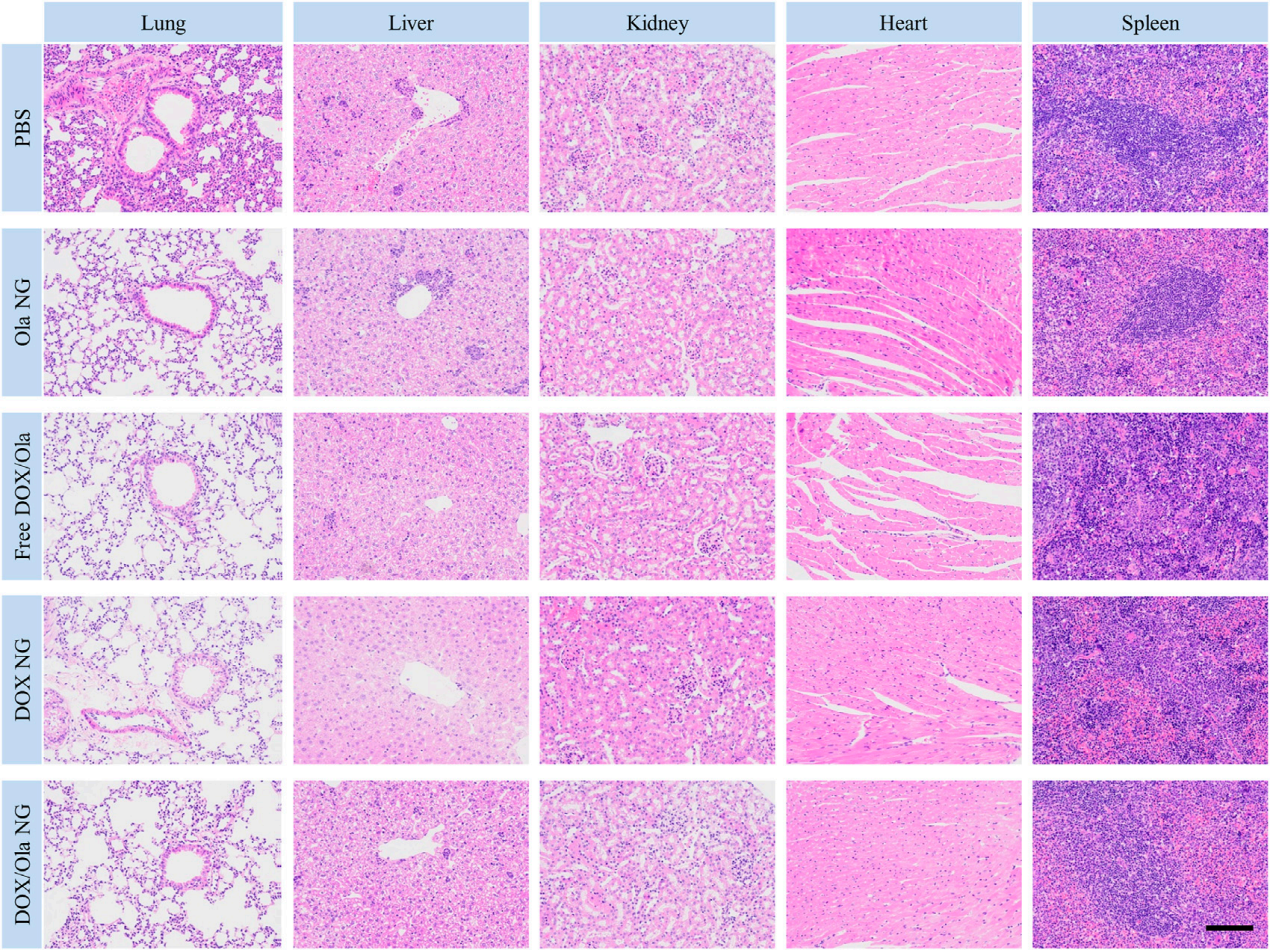
H&E staining of lung, liver, kidney, heart, and spleen specimens after treatment. The scale bar is 100 μm.

It is well known that 4T1 leads to liver and lung metastases (Pulaski and Ostrand-Rosenberg, 2000; da Rocha et al., 2020). Therefore, to evaluate the inhibitory effect of nanomedicine on tumor cell metastasis, H&E slices of organs were studied. As shown in Figure 8, the PBS group has the largest metastases. Compared to the PBS group, the nanomedicine group has smaller metastases. Thus, we show that nanomedicine can achieve similar inhibition with the free drug with negligible physiological toxicity and interfere with the ability of tumor cells to metastasize.

In summary, DOX/Ola can effectively inhibit the progression and metastatic phenotype of 4T1 cells, and the encapsulation of mPEG-polypeptide nanogel can further improve the efficacy of the drug and enhance the drug delivery.

In addition to excellent anti-tumor properties, the insignificant physiological toxicity to organisms is also another key factor for the broad application prospects of nanocarriers. Therefore, to evaluate the biosafety of the nanomedicine, the body weight of the mice was monitored during the treatment period. As shown in the figure, except for the free drug group, other mice showed lower weight fluctuations under the treatment dose, while the free drug group showed a trend of weight loss. The results show that drug delivery *via* nanogels can significantly reduce the side effects of drugs.

The physiological toxicity of the drug to animals also manifests itself in the damage to the organs. At the end of the *in vivo* anti-tumor test model on day18, the tumor-bearing mice were euthanized, and the lung, liver, kidney, heart, and spleen were excised. After fixation in 4% PFA for 1 day, the organs were dehydrated, washed, embedded in paraffin, and cut into 5 μm-thick sections for H&E staining. As shown in Figure 8, the toxicity of free drugs to the heart and liver was observed from the H&E-stained sections. Following treatment with the free drug, the arrangement of myocardial cells was disordered and some muscle fibers were broken. At the same time, no changes in the histopathological morphology was observed in the liver, spleen, lung, and kidney sections. Therefore, free drug caused cardiac damage in tumor-bearing mice, consistent with the reasons for weight loss described earlier.

## Conclusion

In this study, a reduction-responsive cross-linked nanogel mPEG-P (LP-co-LC) was prepared using the ring-opening reaction of amino acid NCA. This promising nanomaterial was used to co-deliver the molecular targeting drug Ola and the chemotherapeutic drug DOX to enhance local cancer treatment. In *vitro* experiments, compared to the Free drug groups, the nanogel preparations showed better cytotoxic and endocytosis effects. In the *in vivo* tests, the 4T1-bearing mouse model showed significant anti-tumor efficacy and migration inhibitory efficacy. Due to the targeted release of nanogels, compared with free drugs, nanogels using the same dose of drugs have a superior biological safety profile. Therefore, reduction-responsive nanogels co-loaded with DOX and Ola have extremely broad prospects for the treatment of breast cancer.

## Data Availability

The original contributions presented in the study are included in the article/Supplementary Material, further inquiries can be directed to the corresponding author.
